# 

**Published:** 1884-01

**Authors:** 


BOOKS.

A Manual of Pathology. Joseph Coates, Glasgow. Philadelphia : Henry C. Lea, Son & Co. Jansen, McClurg & Co.

Insanity in its Medico-Legal Relations. T. Buckham. Philadelphia : Lippincott & Co. Jansen, McClurg & Co.

Venereal Diseases. Bumstead & Taylor, Fifth edition. Philadelphia: H. C. Lea, Son & Co. Jansen, McClurg & Co.

Chemistry, Medical, General, etc. Attfield. Tenth edition. Philadelphia: H. C. Lea, Son & Co. Jansen, McClurg & Co.

Manual of Practical Hygiene. Parkes, Wood's Med. Lib. Sept., 1883 Keener.

Training Schools for Nurses. Notes on 22 Schools. W. G. Thompson New York: Putnams Sons. Jansen, McClurg & Co.

Chemistry, Organic and Inorganic. Bloxam. Fifth edition. Philadelphia: H. C. Lea's Son & Co. Jansen, McClurg & Co.

Hand Book of Therapeutics. Ringer. Tenth edition. New York: Wm. Wood & Co. W. T. Keener.

Materia Medica and Therapeutics. Bartholow. Fifth edition. New York: D. Appleton & Co. Jansen, McClurg & Co.

A Treatise on Syphilis in Children and Infants. P. Diday. Translated from French by G. Whitley. New York: Wm. Wood & Co. W. T Keener.

Bright's Disease of the Kidneys. Henry B. Millard. New York: Wm Wood & Co. W. T. Keener.

Medical Ethics and Medical Etiquette. A Symposium from the Liberal Standpoint. G. P. Putnam's Sons.

Elements of Histology Manual. E. Klein. Philadelphia: H. C. Lea, Son & Co. Jansen, McClurg & Co.

Manual of General Medical Technology. Edward Curtis. New York : Wm. Wood & Co. W. T. Keener.

Physicians' Memorandum Book. Arranged by Joel A. Miner, Ann Arbor, Michigan.

The Physicians' Pocket Day Book. Designed by C. Henri Leonard. Illus Med. Journal Co. Detroit, Mich.

Annual Report of the Supervising Surgeon-General of the Marine Hospital Service.

### PAMPHLETS.

The Study of Neglected Lacerations of the Cervix Uteri and Perineum. Ashby.

Concealed Insanity. Case of Mark Gray. Brower.

A Tracheotomy Tube for Gradual Withdrawal. Hendrix.

The treatment of Syphilis. Sims.

Remarks on Ovariotomy Treatment of Pedicle.

Alcoholic Insanity. Mason.

Antiseptic Treatment of Typhoid Fever. Smythe.

---

PROF. GEO. H. ROHE, of Baltimore, has been delivering a course of lectures upon Hygiene before the Minnesota College Hospital. The lectures have been replete with information, and should have been listened to by the profession of the State, as well as by the class. Dr. Rohe's delivery is quiet, but very effective—his subject matter is beyond criticism. Many of our prominent physicians have been urging his removal to Minnesota, for the practice of his specialty (dermatology), but his engagements with his own college (Physicians and Surgeons of Maryland), may prevent.

Prof. Rohe was tendered a reception by Dr. Stone, Friday evening the 26th ult., meeting all the leading members of the profession of St. Paul, who were unaminous in the expression of their desire that Dr. R. should locate with us permanently. Dr. R. is obliged to cut his visit to St. Paul short, as his engagements at Detroit, during the meeting of the Public Health Association, are imperative.

We wish to congratulate the college upon having secured the course, and the profession at large, upon the prospective addition to its membership.—*Northwestern Lancet.*

